# The α-Synuclein Seeding Amplification Assay for Parkinson’s Disease

**DOI:** 10.3390/ijms26010389

**Published:** 2025-01-04

**Authors:** Ling-Xiao Yi, Eng King Tan, Zhi Dong Zhou

**Affiliations:** 1 National Neuroscience Institute of Singapore, 11 Jalan Tan Tock Seng, Singapore 30843, Singapore; ylxiao9435@hotmail.com; 2 Department of Neurology, Singapore General Hospital, Outram Road, Singapore 169608, Singapore; 3 Signature Research Program in Neuroscience and Behavioral Disorders, Duke-NUS Graduate Medical School Singapore, 8 College Road, Singapore 169857, Singapore

**Keywords:** α-synuclein, biomarker, neurodegeneration, Parkinson’s disease, seeding amplification assay

## Abstract

Parkinson’s disease (PD) is the second most common neurodegenerative disease in the world. Currently, PD is incurable, and the diagnosis of PD mainly relies on clinical manifestations. The central pathological event in PD is the abnormal aggregation and deposition of misfolded α-synuclein (α-Syn) protein aggregates in the Lewy body (LB) in affected brain areas. Behaving as a prion-like seeding, the misfolded α-syn protein can induce and facilitate the aggregation of native unfolded α-Syn protein to aggravate α-Syn protein aggregation, leading to PD progression. Recently, in a blood-based α-Syn seeding amplification assay (SAA), Kluge et al. identified pathological α-Syn seeding activity in PD patients with *Parkin* (PRKN) gene variants. Additionally, pathological α-syn seeding activity was also identified in sporadic PD and PD patients with *Leucine-rich repeat kinase 2* (LRRK2) or *glucocerebrosidase* (GBA) gene variants. Principally, the α-Syn SAA can be used to detect pathological α-Syn seeding activity, which will significantly enhance PD diagnosis, progression monitoring, prognosis prediction, and anti-PD therapy. The significance and future strategies of α-Syn SAA protocol are highlighted and proposed, whereas challenges and limitations of the assay are discussed.

## 1. Introduction

Parkinson’s Disease (PD) is the second most common neurodegenerative disorder that is clinically diagnosed based on motor symptoms (bradykinesia, tremor, rigidity, hypomimia, shuffling gait, difficulty walking, and postural instability) and non-motor symptoms (cognitive decline, autonomous disorders, disrupted sleep, and sensory disturbances) [1,2,3]. The progressive loss of dopaminergic neurons in substantia nigra pars compacta (SNpc), and the deposition of cytoplasmic protein inclusions referred to as Lewy bodies (LBs) in affected brain areas are two pathological hallmarks of PD [4]. Motor symptoms usually occur late in the neurodegenerative process in PD patients, as more than half of striatal dopaminergic neurons are lost when motor symptoms occur [3]. Non-motor symptoms may precede the development of motor symptoms, and thus, can be considered a prodromal state of PD [5]. So far, the exact pathogenesis mechanism behind PD is still unknown. It has been suggested that endogenous dopamine (DA) can be considered an endogenous pathogenic factor for PD [6]. In dopaminergic neurons, DA can undergo auto-oxidation and enzyme-catalyzed metabolism to generate deleterious oxidative DA metabolites, including reactive oxygen species (ROS), DA quinones (DAQs), 3,4-dihydroxyphenylacetaldehyde (DOPAL), and neuromelanin (NM), contributing to the dopaminergic neuron impairment [6,7].

Currently, PD is still an incurable neurodegenerative disease without any therapeutic strategy that could halt or reverse the progressive loss of dopaminergic neurons. The levodopa (L-DOPA) replenishing strategy is the current gold standard for clinical PD treatment to alleviate PD symptoms [8]. Numerous active and passive immunotherapies have exhibited promising therapeutic efficacy in preclinical studies, and many of these are currently undergoing clinical trials [9]. Therefore, an objective and reliable biomarker is urgently required to enhance the diagnostic accuracy of PD in its early stage or prodromal state and monitor its progression. Accumulated evidence suggests that the abnormal deposition and spreading of the misfolded and aggregated α-synuclein (α-Syn) proteins are the central molecular events behind PD pathogenesis [10]. The misfolded and aggregated α-Syn protein is involved in multiple cellular dysfunctions, including the disruption of the autophagy-lysosomal pathway, the dysregulation of mitochondrial function, and the disappearance of dopaminergic neurons [10,11]. The toxicity caused by the aggregated α-Syn protein is closely related to DA and DA oxidative metabolites [6,12]. The overexpression of α-Syn enhances the DA-dependent dopaminergic neuron toxicity [13]. DA-derived metabolites, such as DAQs and DOPAL, can conjugate with α-Syn protein to stabilize the deleterious oligomer form α-Syn protein and enhance α-Syn protein toxicity [14,15,16,17]. Moreover, α-Syn protein promotes the synthesis of NM, an insoluble granular pigment, in dopaminergic neurons [18]. NM generates ROS under oxidative stress, and the interaction between NM and α-Syn is identified to induce neuroinflammation and microglia activation related to α-Syn-associated DA neurodegeneration [7,19,20]. Therefore, the misfolded and aggregated form of α-syn protein could be considered a vital pathogenic biomarker in PD.

## 2. Physical Function, Structure, and Pathological Role of α-Syn Protein

The α-Syn, encoded by the *SNCA* gene, is a small soluble protein comprising 140 amino acids with three distinct domains: an *N*-terminal domain (1–60 amino acid residues), a non-amyloid-β component (NAC) domain (61–95 amino acid residues), and a C-terminal domain (96–140 amino acid residues) [21]. The *N*-terminal domain is positively charged and characterized by an amphipathic lysine-rich amino terminus that serves as the membrane anchor region of α-Syn [21]. The NAC domain is hydrophobic and constitutes the amino acid segments that are essential in fibril formation and aggregation, which has been identified as the most aggregation-prone region [21]. The C-terminal domain is a negatively charged tail that does not contain secondary structures, including ten glutamate and five aspartate residues, characterized by disease-promoting regions and involved in chaperone-like activity [22].

The α-syn is abundant in the brains and exists in erythrocytes, platelets, and other tissues [23]. In the brains, α-Syn is primarily expressed in neuronal cells, located in the pre-synaptic terminal and probably bound to the membrane of synaptic vesicles [24]. It exists in the dynamic equilibrium in the unfolded form in the cytosol and an α helical-rich form when bound to membranes, which is thought to play a vital role in modulating the stability of the membrane, altering membrane trafficking via vesicular transport and promoting the assembly of soluble *N*-ethylmaleimide-sensitive factor attachment protein receptor complex (SNARE) [25,26]. Native α-Syn mainly exists as an unfolded monomer, dimer, or tetramer. However, under disease conditions, the native α-Syn can aggregate and misfold into pathological α-syn oligomers, also known as aggregates, to increase membrane permeability and disrupt membrane integrity [27]. Furthermore, the α-Syn oligomers can further polymerize to form α-Syn fibrils and accumulate in the brain, peripheral tissues, and biofluids [28]. The aggregation of α-Syn fibrils is believed to be the pathological hallmark of synucleinopathies, including PD, Dementia with Lewy Bodies (DLB), and multiple system atrophy (MSA), based on the cellular predilections and neuroanatomical distributions [11,29].

## 3. Detection of Pathological α-Syn Aggregates by Seeding Amplification Assays (SAAs) in PD Patients

### 3.1. Principle of α-Syn SAAs

The α-syn seed amplification assays (SAAs), previously known as the real-time quaking-induced conversion (RT-QuIC) and protein misfolding cyclic amplification (PMCA), have shown promise in detecting pathological α-Syn aggregates in different biometrics from living patients with synucleinopathies, with relatively higher specificity and sensitivity [30,31,32,33,34]. The α-Syn SAAs use the intrinsic self-propagating nature of misfolded α-syn oligomers or aggregates as seeds to amplify themselves in vitro. Amplification is obtained by adding excessive monomeric α-syn as a reactant into the tested samples. These α-syn oligomers or aggregates in the tested samples will recruit reactants and elongate into fibrils [30]. Subsequently, the elongated fibrils will be fragmented into new fibrils by shaking the samples, whereas the newly created fibrils again grow by recruiting monomeric reactants, and this cycle continues [30]. Hence, the presence of α-Syn seeds can be monitored in real-time by detecting the fluorescent intensity of thioflavin T (ThT), which can be detected upon its binding to the fibril and allows for the use of total fluorescence intensity as a readout once the seeds are sufficiently amplified [30]. According to the kinetic parameters and filament structure of α-Syn seeds, PD patients can be differentiated from healthy individuals or related synucleinopathies [31,35,36,37,38].

### 3.2. Detection of Pathological α-Syn by SAAs in PD Patients

So far, most PD cases are sporadic PD (SPD), and their exact pathogenesis is unclear. The pathogenesis of familial PD (FPD) is closely related to mutations in multiple pathogenic genes, including *Leucine-rich repeat kinase 2* (LRRK2), *PTEN-induced kinase 1* (PINK1), *Parkin* (PRKN), *glucocerebrosidase* (GBA), *DJ-1*, and SNCA [6]. The positive α-Syn seeding activity has been detected in both SPD patients and FPD patients from multiple biometrics by performing SAAs, including skin, gut, submandibular gland, olfactory mucosa, cerebrospinal fluid (CSF), and blood [32,34,38,39,40,41,42,43,44,45,46,47,48]. Among these biometrics, CSF represents a potentially reliable source for neurodegenerative disease [49]. Using SAA in CSF, a previous study demonstrated that the prevalence of α-Syn seeding activity in SPD patients (91%) and FPD patients carrying *GBA* mutations (87%) was higher than that of FPD patients carrying *LRRK2* mutations (78%) or recessive heterozygous mutations (59%) [46]. FPD patients carrying bi-allelic mutations in recessively inherited genes like *PINK1* and *PRKN* did not present positive α-Syn seeding activity [46]. Similar patterns were observed in a more recent study, which demonstrated that the prevalence of positive α-Syn seeding activity was higher in FPD patients carrying *GBA* mutations, followed closely by the SPD patients, and a substantially lower prevalence was identified in FPD patients carrying *LRRK2* mutations [32].

Moreover, due to the limited availability of CSF samples, blood-based biomarkers have emerged in numeric studies in recent years. Previous studies have proved that higher levels of α-Syn in plasma and serum have been found to positively correlate to motor deficits and cognitive decline in PD patients [50,51,52]. Meanwhile, PRKN, a PD-relevant ubiquitin E3 ligase, was found to be involved in the modulation of mitochondria function and autophagy-lysosomal pathway [53,54]. Mutations in *PRKN* are recognized as the most frequent pathological causes of autosomal recessive early-onset PD [55]. In a recent study involving 32 participants (9 idiopathic PD patients, 13 PD patients with pathogenic biallelic PRKN variants, and 10 healthy controls), Kluge et al. evaluated the prevalence of α-Syn seeding activity by blood-based α-Syn SAAs [40]. Through analysis of neuron-derived extracellular vesicles (NEVs) extracted from patient blood samples, the positive α-Syn seeding activity was confirmed in 8 of 9 idiopathic PD patients, 8 of 13 PD patients with pathogenic biallelic *PRKN* variants, and none of the healthy controls [40]. These findings, for the first time, indicated that α-Syn seeding activity could be a consistent pathological feature of PRKN-linked PD, supporting that α-Syn seeding activity monitored by NEVs-dependent SAA protocol can be a promising biomarker for PD [40].

## 4. Significance, Challenges, and Future Directions

The oligomeric α-Syn, the earlier stage of α-Syn aggregation, is widely distributed in multiple cortical and subcortical regions, contributing to synaptic dysregulation, dopaminergic neurodegeneration, motor deficits, and cognitive impairments in PD patients [56,57]. Therefore, detecting oligomeric α-Syn could be considered a routine strategy to distinguish PD patients from healthy individuals or related synucleinopathies in the prodromal or early stage. Nevertheless, multiple factors, such as the distribution of oligomeric α-Syn, gene mutation, post-translational modifications, and exposure to mental heavy, have positive or negative effects on the α-Syn seeding activity, which needs to be re-assessed, re-evaluated, and re-addressed in future studies for precision diagnosis.

Until now, the presence of α-Syn seeds has been detected in various biological fluids, especially CSF and peripheral blood. According to the gut-to-brain axis hypothesis, the misfolded or toxic oligomeric α-Syn originates from the periphery enteric plexus or erythrocytes. The erythrocytes-derived and periphery-derived α-Syn oligomers can spread from the gut to the brain via the enteric nervous system, vagus nerve, and glossopharyngeal nerve [58,59]. Meanwhile, previous studies have shown that the aberrant deposition of α-Syn in the intestinal tract was earlier than the loss of dopaminergic neurons and the occurrence of motor symptoms of PD [60]. Nevertheless, the presence of oligomeric α-Syn in specific body tissues and the occurrence of PD clinical features has not been fully determined, which needs to be emphasized, evaluated, and addressed in future studies to provide scientific insight for further application of α-Syn SAAs-based strategy for PD precision diagnosis.

Mutations in *SNCA*, including missense and multiplication mutations, can cause early-onset autosomal-dominant PD [61]. Six missense point mutations in the *N*-terminal domain, including A53T, A30P, A53E, E46K, H50Q, and G51D, have been strongly associated with autosomal dominant familial PD, which might be related to their capacities to influence the oligomerization or fibrillation of α-Syn protein [62,63,64,65]. PD patients affected by *SNCA* point mutations present a clinical phenotype similar to those with sporadic PD, with an earlier age at onset, and often suffer from dementia and autonomic disturbances [66]. Recent SAAs have demonstrated that the A53T mutation has a more robust seeding activity than the wild-type (WT) protein [67]. Moreover, PD patients with *SNCA* duplication exhibit a typical late-onset PD phenotype, and PD patients with *SNCA* triplication have been reported to cause hereditary early-onset PD phenotype with dementia [68,69]. It has been demonstrated that the α-Syn seeding activities in the CSF from PD patients with triplication were more robust than those of the WT PD patients [70]. Other PD-related genes, such as *LRRK2*, *GBA1*, *DJ1*, *PINK1*, and *PRKN*, can significantly influence the misfolding and aggregation of α-Syn [6]. Among them, *PRKN*, *PINK1*, and *DJ-1* variants are usually found in individuals with autosomal recessive early-onset PD [71]. In contrast, variants in *GBA1* and *LRRK2* are widely accepted as the two most crucial genetic risk factors associated with PD [72]. The *GBA* variants, such as the L444P and N370S variants and *LRRK2* variants, especially the G2019S variant, can remarkably promote α-Syn aggregation [72,73,74]. Recent studies highlight that PD patients carrying *GBA* and *LRRK2* variants have higher seeding activity in CSF than those carrying *PINK1* and *PRKN* [46]. So far, the effects of PD-related pathogenic genes and their variants on α-Syn seeding activity have not been thoroughly investigated. Genetic tests, such as next-generation sequencing, have been used to identify pathogenic variants in PD patients [75]. Hence, screening the pathogenic variants by multiple genetic tests could be considered an adjunct measurement to improve the sensitivity and accuracy of SAA.

α-Syn is subjected to extensive post-translational modification, such as truncation, phosphorylation, ubiquitination, nitration, and *O*-GlcNAcylation, which could influence its aggregation and cytotoxicity. The C-terminal truncated α-Syn produced by aberrant proteolysis has been detected in the brains of healthy individuals and PD patients, and it is thought to be associated with the formation of α-Syn aggregates [76]. Previous studies have shown that C-terminal truncated fragments, α-Syn (1–108) and α-Syn (1–124), aggregate faster than the full-length α-Syn protein [77]. Recent SAA has proved that the PD-related C-terminal 123–140 and 104–140 truncations of α-Syn present higher seeding activity than the WT α-Syn [67]. Further mechanistic studies revealed that C-terminal truncations promote the conversion of monomers to aggregated forms of α-Syn, enhance autocatalytic aggregation on existing fibrils, and interfere with the interactions between the *N*-terminal of α-Syn and the membrane [67,76]. These findings demonstrated that the truncation of α-Syn affects their seeding activity and aggregating properties. In addition, truncation, phosphorylation, ubiquitination, nitration, and *O*-GlcNAcylation of α-Syn have been reported to influence α-Syn aggregation and fibril formation at various degrees [78,79,80,81,82,83,84]. The effects of these post-translational modifications on the α-Syn seeding activity have not been comprehensively evaluated, which needs to be emphasized and addressed in future studies. Moreover, proteomics techniques, particularly those based on high-resolution mass spectrometry, have been rapidly developed as a preferred method to determine relative changes in post-translational modifications and protein abundance [85]. Thus, screening and monitoring post-translational modifications via multiple proteomic techniques may enhance the sensitivity and accuracy of SAA, which need to be assessed, evaluated, and addressed in future studies.

Numerous epidemiological studies have correlated exposure to metal ions with the onset and development of PD [86]. The pathogenic effects of metal in PD patients are associated with their capacities to elevate ROS levels and enhance aberrant α-Syn aggregation in the brain [87,88]. It was previously reported that divalent or trivalent metal ions, particularly aluminum (III), copper (II), iron (II), cobalt (III), and manganese (II), significantly accelerated the rate of α-Syn aggregation and fibril formation [89]. Recent studies indicated that bivalent metal ions, including zinc (II), copper (II), calcium (II), and manganese (II), accelerate the α-Syn fibrillation and form different amyloid-competent conformations, suggesting that α-Syn exposure to these mental ions have a higher seeding activity [90,91]. These α-Syn fibrils formed in the presence of different bivalent metal ions have distinct kinetics, size, morphology, secondary structure, and cytotoxicity, as well as the charge status of bivalent metal ions (measured by the native nanoelectrospray ionization ion mobility-mass spectrometry) was remarkably reduced when bound to the α-Syn [90,91]. Moreover, the bivalent metal ions have also been reported to significantly promote the aggregation of mutant α-Syn protein. For instance, in the presence of copper (II) ion, the mutants G51D α-Syn protein have higher aggregation kinetics and fibril formation rate than WT α-Syn protein [92]. Nevertheless, further studies are warranted to evaluate the pathogenic effects of other trivalent or heavy metal ions on α-Syn aggregation and fibril formation. Strategies that determine the morphology and structure of fibrils formed by α-Syn aggregates and the charge status of metal ions could be used as an adjunct measurement to SAA, which also needs to be addressed in future studies.

## 5. Conclusions

The α-Syn seeding activity detected by SAA is a promising strategy for distinguishing PD patients from healthy individuals and other synucleinopathies. Further studies are necessary to systematically evaluate the distribution of α-Syn seeds in different biofluids. After that, multiple strategies to screen for pathogenic variants, post-translational modifications, and environmental toxins can be used as adjunct measurements to improve the sensitivity and accuracy of the α-Syn SAA test.

## References

[1] Löhle M., Storch A., Reichmann H. (2009). Beyond tremor and rigidity: Non-motor features of Parkinson’s disease. J. Neural Transm. Park. Dis. Dement. Sect..

[2] Postuma R.B., Poewe W., Litvan I., Lewis S., Lang A.E., Halliday G., Goetz C.G., Chan P., Slow E., Seppi K. (2018). Validation of the MDS clinical diagnostic criteria for Parkinson’s disease. Mov. Disord..

[3] Dauer W., Przedborski S. (2003). Parkinson’s Disease: Mechanisms and Models. Neuron.

[4] Joseph J., Eng King T. (2020). Parkinson’s disease: Etiopathogenesis and treatment. J. Neurol. Neurosurg. Psychiatry.

[5] Hustad E., Aasly J.O. (2020). Clinical and Imaging Markers of Prodromal Parkinson’s Disease. Front. Neurol..

[6] Zhou Z.D., Yi L.X., Wang D.Q., Lim T.M., Tan E.K. (2023). Role of dopamine in the pathophysiology of Parkinson’s disease. Transl. Neurodegener..

[7] Li J., Yang J., Zhao P., Li S., Zhang R., Zhang X., Liu D., Zhang B. (2012). Neuromelanin enhances the toxicity of α-synuclein in SK-N-SH cells. J. Neural Transm. Park. Dis. Dement. Sect..

[8] Tomlinson C.L., Stowe R., Patel S., Rick C., Gray R., Clarke C.E. (2010). Systematic review of levodopa dose equivalency reporting in Parkinson’s disease. Mov. Disord..

[9] Henriquez G., Narayan M. (2023). Targeting α-synuclein aggregation with immunotherapy: A promising therapeutic approach for Parkinson’s disease. Explor. Neuroprotective Ther..

[10] Calabresi P., Mechelli A., Natale G., Volpicelli-Daley L., Di Lazzaro G., Ghiglieri V. (2023). Alpha-synuclein in Parkinson’s disease and other synucleinopathies: From overt neurodegeneration back to early synaptic dysfunction. Cell Death Dis..

[11] Wells C., Brennan S., Keon M., Ooi L. (2021). The role of amyloid oligomers in neurodegenerative pathologies. Int. J. Biol. Macromol..

[12] Sivakumar P., Nagashanmugam K.B., Priyatharshni S., Lavanya R., Prabhu N., Ponnusamy S. (2023). Review on the interactions between dopamine metabolites and α-Synuclein in causing Parkinson’s disease. Neurochem. Int..

[13] Bisaglia M., Greggio E., Maric D., Miller D.W., Cookson M.R., Bubacco L. (2010). α-Synuclein overexpression increases dopamine toxicity in BE(2)-M17 cells. BMC Neurosci..

[14] Conway K.A., Rochet J.C., Bieganski R.M., Lansbury P.T. (2001). Kinetic stabilization of the alpha-synuclein protofibril by a dopamine-alpha-synuclein adduct. Science.

[15] Mor D.E., Tsika E., Mazzulli J.R., Gould N.S., Kim H., Daniels M.J., Doshi S., Gupta P., Grossman J.L., Tan V.X. (2017). Dopamine induces soluble α-synuclein oligomers and nigrostriatal degeneration. Nat. Neurosci..

[16] Bisaglia M., Tosatto L., Munari F., Tessari I., de Laureto P.P., Mammi S., Bubacco L. (2010). Dopamine quinones interact with alpha-synuclein to form unstructured adducts. Biochem. Biophys. Res. Commun..

[17] Follmer C., Coelho-Cerqueira E., Yatabe-Franco D.Y., Araujo G.D., Pinheiro A.S., Domont G.B., Eliezer D. (2015). Oligomerization and Membrane-binding Properties of Covalent Adducts Formed by the Interaction of α-Synuclein with the Toxic Dopamine Metabolite 3,4-Dihydroxyphenylacetaldehyde (DOPAL). J. Biol. Chem..

[18] Xu S., Chan P. (2015). Interaction between Neuromelanin and Alpha-Synuclein in Parkinson’s Disease. Biomolecules.

[19] Bustamante J., Bredeston L., Malanga G., Mordoh J. (1993). Role of melanin as a scavenger of active oxygen species. Pigment. Cell Res..

[20] Knörle R. (2018). Neuromelanin in Parkinson’s Disease: From Fenton Reaction to Calcium Signaling. Neurotox. Res..

[21] Fusco G., De Simone A., Gopinath T., Vostrikov V., Vendruscolo M., Dobson C.M., Veglia G. (2014). Direct observation of the three regions in α-synuclein that determine its membrane-bound behaviour. Nat. Commun..

[22] Kim T.D., Paik S.R., Yang C.H. (2002). Structural and functional implications of C-terminal regions of alpha-synuclein. Biochemistry.

[23] Del Tredici K., Braak H. (2016). Sporadic Parkinson’s disease: Development and distribution of α-synuclein pathology. Neuropathol. Appl. Neurobiol..

[24] Brás I.C., Outeiro T.F. (2021). Alpha-Synuclein: Mechanisms of Release and Pathology Progression in Synucleinopathies. Cells.

[25] Burré J., Sharma M., Tsetsenis T., Buchman V., Etherton M.R., Südhof T.C. (2010). Alpha-synuclein promotes SNARE-complex assembly in vivo and in vitro. Science.

[26] Sun J., Wang L., Bao H., Premi S., Das U., Chapman E.R., Roy S. (2019). Functional cooperation of α-synuclein and VAMP2 in synaptic vesicle recycling. Proc. Natl. Acad. Sci. USA.

[27] Suzuki M., Sango K., Wada K., Nagai Y. (2018). Pathological role of lipid interaction with α-synuclein in Parkinson’s disease. Neurochem. Int..

[28] Brundin P., Melki R., Kopito R. (2010). Prion-like transmission of protein aggregates in neurodegenerative diseases. Nat. Rev. Mol. Cell Biol..

[29] Halliday G.M., Holton J.L., Revesz T., Dickson D.W. (2011). Neuropathology underlying clinical variability in patients with synucleinopathies. Acta Neuropathol..

[30] Vaneyck J., Yousif T.A., Segers-Nolten I., Blum C., Claessens M.M.A.E. (2023). Quantitative Seed Amplification Assay: A Proof-of-Principle Study. J. Phys. Chem. B.

[31] Fernandes Gomes B., Farris C.M., Ma Y., Concha-Marambio L., Lebovitz R., Nellgård B., Dalla K., Constantinescu J., Constantinescu R., Gobom J. (2023). α-Synuclein seed amplification assay as a diagnostic tool for parkinsonian disorders. Park. Relat. Disord..

[32] Siderowf A., Concha-Marambio L., Lafontant D.E., Farris C.M., Ma Y., Urenia P.A., Nguyen H., Alcalay R.N., Chahine L.M., Foroud T. (2023). Assessment of heterogeneity among participants in the Parkinson’s Progression Markers Initiative cohort using α-synuclein seed amplification: A cross-sectional study. Lancet Neurol..

[33] Manne S., Kondru N., Jin H., Serrano G.E., Anantharam V., Kanthasamy A., Adler C.H., Beach T.G., Kanthasamy A.G. (2020). Blinded RT-QuIC Analysis of α-Synuclein Biomarker in Skin Tissue From Parkinson’s Disease Patients. Mov. Disord..

[34] Wang Z., Becker K., Donadio V., Siedlak S., Yuan J., Rezaee M., Incensi A., Kuzkina A., Orrú C.D., Tatsuoka C. (2021). Skin α-Synuclein Aggregation Seeding Activity as a Novel Biomarker for Parkinson Disease. JAMA Neurol..

[35] Khedmatgozar C.R., Holec S.A.M., Woerman A.L. (2024). The role of α-synuclein prion strains in Parkinson’s disease and multiple system atrophy. PLoS Pathog..

[36] Shahnawaz M., Mukherjee A., Pritzkow S., Mendez N., Rabadia P., Liu X., Hu B., Schmeichel A., Singer W., Wu G. (2020). Discriminating α-synuclein strains in Parkinson’s disease and multiple system atrophy. Nature.

[37] Yang Y., Shi Y., Schweighauser M., Zhang X., Kotecha A., Murzin A.G., Garringer H.J., Cullinane P.W., Saito Y., Foroud T. (2022). Structures of α-synuclein filaments from human brains with Lewy pathology. Nature.

[38] De Luca C.M.G., Elia A.E., Portaleone S.M., Cazzaniga F.A., Rossi M., Bistaffa E., De Cecco E., Narkiewicz J., Salzano G., Carletta O. (2019). Efficient RT-QuIC seeding activity for α-synuclein in olfactory mucosa samples of patients with Parkinson’s disease and multiple system atrophy. Transl. Neurodegener..

[39] Chen D.-D., Jiao L., Huang Y., Xiao K., Gao L.-P., Chen C., Shi Q., Dong X.-P. (2022). Application of α-Syn Real-Time Quaking-Induced Conversion for Brain and Skin Specimens of the Chinese Patients With Parkinson’s Disease. Front. Aging Neurosci..

[40] Kluge A., Borsche M., Streubel-Gallasch L., Gül T., Schaake S., Balck A., Prasuhn J., Campbell P., Morris H.R., Schapira A.H. (2024). α-Synuclein Pathology in PRKN-Linked Parkinson’s Disease: New Insights from a Blood-Based Seed Amplification Assay. Ann. Neurol..

[41] Kluge A., Schaeffer E., Bunk J., Sommerauer M., Röttgen S., Schulte C., Roeben B., von Thaler A.-K., Welzel J., Lucius R. (2024). Detecting Misfolded α-Synuclein in Blood Years before the Diagnosis of Parkinson’s Disease. Mov. Disord..

[42] Annika K., Carmen K., Kristina K., Katja S., Julius W., Sebastian H., Thilo W., Martina B., Ralph L., Sarah Kim B. (2024). Alpha-synuclein distribution and seeding activity in rectal biopsies in Parkinson’s disease. medRxiv.

[43] Fenyi A., Leclair-Visonneau L., Clairembault T., Coron E., Neunlist M., Melki R., Derkinderen P., Bousset L. (2019). Detection of alpha-synuclein aggregates in gastrointestinal biopsies by protein misfolding cyclic amplification. Neurobiol. Dis..

[44] Shin C., Han J.Y., Kim S.I., Park S.H., Yang H.K., Lee H.J., Kong S.H., Suh Y.S., Kim H.J., Choi Y.P. (2022). In vivo and autopsy validation of alpha-synuclein seeding activity using RT-QuIC assay in the gastrointestinal tract of patients with Parkinson’s disease. Park. Relat. Disord..

[45] Brockmann K., Lerche S., Baiardi S., Rossi M., Wurster I., Quadalti C., Roeben B., Mammana A., Zimmermann M., Hauser A.K. (2024). CSF α-synuclein seed amplification kinetic profiles are associated with cognitive decline in Parkinson’s disease. NPJ Park. Dis..

[46] Brockmann K., Quadalti C., Lerche S., Rossi M., Wurster I., Baiardi S., Roeben B., Mammana A., Zimmermann M., Hauser A.-K. (2021). Association between CSF alpha-synuclein seeding activity and genetic status in Parkinson’s disease and dementia with Lewy bodies. Acta Neuropathol. Commun..

[47] Manne S., Kondru N., Jin H., Anantharam V., Huang X., Kanthasamy A., Kanthasamy A.G. (2020). α-Synuclein real-time quaking-induced conversion in the submandibular glands of Parkinson’s disease patients. Mov. Disord..

[48] Garrido A., Fairfoul G., Tolosa E.S., Martí M.J., Green A., Barcelona LRRK2 Study Group (2019). α-synuclein RT-QuIC in cerebrospinal fluid of LRRK2-linked Parkinson’s disease. Ann. Clin. Transl. Neurol..

[49] Tao M., Dou K., Xie Y., Hou B., Xie A. (2022). The associations of cerebrospinal fluid biomarkers with cognition, and rapid eye movement sleep behavior disorder in early Parkinson’s disease. Front. Neurosci..

[50] Wang L., Wang G., Duan Y., Wang F., Lin S., Zhang F., Li H., Li A., Li H. (2019). A Comparative Study of the Diagnostic Potential of Plasma and Erythrocytic α-Synuclein in Parkinson’s Disease. Neurodegener. Dis..

[51] Chang C.W., Yang S.Y., Yang C.C., Chang C.W., Wu Y.R. (2019). Plasma and Serum Alpha-Synuclein as a Biomarker of Diagnosis in Patients With Parkinson’s Disease. Front. Neurol..

[52] Ng A.S.L., Tan Y.J., Lu Z., Ng E.Y.L., Ng S.Y.E., Chia N.S.Y., Setiawan F., Xu Z., Tay K.Y., Prakash K.M. (2019). Plasma alpha-synuclein detected by single molecule array is increased in PD. Ann. Clin. Transl. Neurol..

[53] Esteves A.R., Arduíno D.M., Silva D.F.F., Oliveira C.R., Cardoso S.M. (2011). Mitochondrial Dysfunction: The Road to Alpha-Synuclein Oligomerization in PD. Park. Dis..

[54] Truban D., Hou X., Caulfield T.R., Fiesel F.C., Springer W. (2017). PINK1, Parkin, and Mitochondrial Quality Control: What can we Learn about Parkinson’s Disease Pathobiology?. J. Park. Dis..

[55] Wasner K., Grünewald A., Klein C. (2020). Parkin-linked Parkinson’s disease: From clinical insights to pathogenic mechanisms and novel therapeutic approaches. Neurosci. Res..

[56] Surmeier D.J., Obeso J.A., Halliday G.M. (2017). Selective neuronal vulnerability in Parkinson disease. Nat. Rev. Neurosci..

[57] Sekiya H., Tsuji A., Hashimoto Y., Takata M., Koga S., Nishida K., Futamura N., Kawamoto M., Kohara N., Dickson D.W. (2022). Discrepancy between distribution of alpha-synuclein oligomers and Lewy-related pathology in Parkinson’s disease. Acta Neuropathol. Commun..

[58] Mulak A., Bonaz B. (2015). Brain-gut-microbiota axis in Parkinson’s disease. World J. Gastroenterol..

[59] Schmitt V., Masanetz R.K., Weidenfeller M., Ebbinghaus L.S., Süß P., Rosshart S.P., von Hörsten S., Zunke F., Winkler J., Xiang W. (2023). Gut-to-brain spreading of pathology in synucleinopathies: A focus on molecular signalling mediators. Behav. Brain Res..

[60] Chen Q.-Q., Haikal C., Li W., Li M.-T., Wang Z.-Y., Li J.-Y. (2018). Age-dependent alpha-synuclein accumulation and aggregation in the colon of a transgenic mouse model of Parkinson’s disease. Transl. Neurodegener..

[61] Srinivasan E., Chandrasekhar G., Chandrasekar P., Anbarasu K., Vickram A.S., Karunakaran R., Rajasekaran R., Srikumar P.S. (2021). Alpha-Synuclein Aggregation in Parkinson’s Disease. Front. Med..

[62] Conway K.A., Lee S.J., Rochet J.C., Ding T.T., Williamson R.E., Lansbury P.T. (2000). Acceleration of oligomerization, not fibrillization, is a shared property of both alpha-synuclein mutations linked to early-onset Parkinson’s disease: Implications for pathogenesis and therapy. Proc. Natl. Acad. Sci. USA.

[63] Lázaro D.F., Rodrigues E.F., Langohr R., Shahpasandzadeh H., Ribeiro T., Guerreiro P., Gerhardt E., Kröhnert K., Klucken J., Pereira M.D. (2014). Systematic comparison of the effects of alpha-synuclein mutations on its oligomerization and aggregation. PLoS Genet..

[64] Boyer D.R., Li B., Sun C., Fan W., Sawaya M.R., Jiang L., Eisenberg D.S. (2019). Structures of fibrils formed by α-synuclein hereditary disease mutant H50Q reveal new polymorphs. Nat. Struct. Mol. Biol..

[65] Rutherford N.J., Giasson B.I. (2015). The A53E α-synuclein pathological mutation demonstrates reduced aggregation propensity in vitro and in cell culture. Neurosci. Lett..

[66] Spira P.J., Sharpe D.M., Halliday G., Cavanagh J., Nicholson G.A. (2001). Clinical and pathological features of a parkinsonian syndrome in a family with an Ala53Thr α-synuclein mutation. Ann. Neurol..

[67] Ohgita T., Namba N., Kono H., Shimanouchi T., Saito H. (2022). Mechanisms of enhanced aggregation and fibril formation of Parkinson’s disease-related variants of α-synuclein. Sci. Rep..

[68] Chartier-Harlin M.-C., Kachergus J., Roumier C., Mouroux V., Douay X., Lincoln S., Levecque C., Larvor L., Andrieux J., Hulihan M. (2004). α-synuclein locus duplication as a cause of familial Parkinson’s disease. Lancet.

[69] Singleton A., Farrer M., Johnson J., Singleton A., Hague S., Kachergus J., Hulihan M., Peuralinna T., Dutra A., Nussbaum R. (2003). α-Synuclein locus triplication causes Parkinson’s disease. Science.

[70] Wurster I., Quadalti C., Rossi M., Hauser A.-K., Deuschle C., Schulte C., Waniek K., Lachmann I., la Fougere C., Doppler K. (2022). Linking the phenotype of SNCA Triplication with PET-MRI imaging pattern and alpha-synuclein CSF seeding. NPJ Park. Dis..

[71] Milanowski Ł.M., Lindemann J.A., Hoffman-Zacharska D., Soto-Beasley A.I., Barcikowska M., Boczarska-Jedynak M., Deutschlander A., Kłodowska G., Dulski J., Fedoryshyn L. (2021). Frequency of mutations in PRKN, PINK1, and DJ1 in Patients With Early-Onset Parkinson Disease from neighboring countries in Central Europe. Park. Relat. Disord..

[72] Smith L.J., Lee C.Y., Menozzi E., Schapira A.H.V. (2022). Genetic variations in GBA1 and LRRK2 genes: Biochemical and clinical consequences in Parkinson disease. Front. Neurol..

[73] Blandini F., Cilia R., Cerri S., Pezzoli G., Schapira A.H.V., Mullin S., Lanciego J.L. (2019). Glucocerebrosidase mutations and synucleinopathies: Toward a model of precision medicine. Mov. Disord..

[74] Bieri G., Brahic M., Bousset L., Couthouis J., Kramer N.J., Ma R., Nakayama L., Monbureau M., Defensor E., Schüle B. (2019). LRRK2 modifies α-syn pathology and spread in mouse models and human neurons. Acta Neuropathol..

[75] Hackl M., Cook L., Wetherill L., Walsh L.E., Delk P., De León R., Carbonell J., Vicioso R.C., Hodges P.D. (2023). Readiness for Parkinson’s disease genetic testing and counseling in patients and their relatives in urban settings in the Dominican Republic. NPJ Park. Dis..

[76] Zhang C., Pei Y., Zhang Z., Xu L., Liu X., Jiang L., Pielak G.J., Zhou X., Liu M., Li C. (2022). C-terminal truncation modulates α-Synuclein’s cytotoxicity and aggregation by promoting the interactions with membrane and chaperone. Commun. Biol..

[77] Hoyer W., Cherny D., Subramaniam V., Jovin T.M. (2004). Impact of the Acidic C-Terminal Region Comprising Amino Acids 109−140 on α-Synuclein Aggregation in Vitro. Biochemistry.

[78] Hejjaoui M., Haj-Yahya M., Kumar K.S.A., Brik A., Lashuel H.A. (2011). Towards Elucidation of the Role of Ubiquitination in the Pathogenesis of Parkinson’s Disease with Semisynthetic Ubiquitinated α-Synuclein. Angew. Chem. Int. Ed. Engl..

[79] Zhang J., Li X., Li J.-D. (2019). The Roles of Post-translational Modifications on α-Synuclein in the Pathogenesis of Parkinson’s Diseases. Front. Neurosci..

[80] Anderson J.P., Walker D.E., Goldstein J.M., de Laat R., Banducci K., Caccavello R.J., Barbour R., Huang J., Kling K., Lee M. (2006). Phosphorylation of Ser-129 Is the Dominant Pathological Modification of alpha-Synuclein in Familial and Sporadic Lewy Body Disease. J. Biol. Chem..

[81] Paleologou K.E., Oueslati A., Shakked G., Rospigliosi C.C., Kim H.Y., Lamberto G.R., Fernandez C.O., Schmid A., Chegini F., Gai W.P. (2010). Phosphorylation at S87 is enhanced in synucleinopathies, inhibits alpha-synuclein oligomerization, and influences synuclein-membrane interactions. J. Neurosci..

[82] Hodara R., Norris E.H., Giasson B.I., Mishizen-Eberz A.J., Lynch D.R., Lee V.M., Ischiropoulos H. (2004). Functional consequences of alpha-synuclein tyrosine nitration: Diminished binding to lipid vesicles and increased fibril formation. J. Biol. Chem..

[83] Haj-Yahya M., Fauvet B., Herman-Bachinsky Y., Hejjaoui M., Bavikar S.N., Karthikeyan S.V., Ciechanover A., Lashuel H.A., Brik A. (2013). Synthetic polyubiquitinated α-Synuclein reveals important insights into the roles of the ubiquitin chain in regulating its pathophysiology. Proc. Natl. Acad. Sci. USA.

[84] Levine P.M., Galesic A., Balana A.T., Mahul-Mellier A.L., Navarro M.X., De Leon C.A., Lashuel H.A., Pratt M.R. (2019). α-Synuclein O-GlcNAcylation alters aggregation and toxicity, revealing certain residues as potential inhibitors of Parkinson’s disease. Proc. Natl. Acad. Sci. USA.

[85] Holtz A., Basisty N., Schilling B. (2021). Quantification and Identification of Post-Translational Modifications Using Modern Proteomics Approaches. Quantitative Methods in Proteomics.

[86] Bjorklund G., Stejskal V., Urbina M.A., Dadar M., Chirumbolo S., Mutter J. (2018). Metals and Parkinson’s Disease: Mechanisms and Biochemical Processes. Curr. Med. Chem..

[87] Zhang Y.-Y., Li X.-S., Ren K.-D., Peng J., Luo X.-J. (2023). Restoration of metal homeostasis: A potential strategy against neurodegenerative diseases. Ageing Res. Rev..

[88] Santner A., Uversky V.N. (2010). Metalloproteomics and metal toxicology of α-synuclein. Metallomics.

[89] Uversky V.N., Li J., Fink A.L. (2001). Metal-triggered structural transformations, aggregation, and fibrillation of human alpha-synuclein. A possible molecular NK between Parkinson’s disease and heavy metal exposure. J. Biol. Chem..

[90] Atarod D., Mamashli F., Ghasemi A., Moosavi-Movahedi F., Pirhaghi M., Nedaei H., Muronetz V., Haertlé T., Tatzelt J., Riazi G. (2022). Bivalent metal ions induce formation of α-synuclein fibril polymorphs with different cytotoxicities. Sci. Rep..

[91] Byrd E.J., Wilkinson M., Radford S.E., Sobott F. (2023). Taking Charge: Metal Ions Accelerate Amyloid Aggregation in Sequence Variants of α-Synuclein. J. Am. Soc. Mass Spectrom..

[92] Ranjan P., Ghosh D., Yarramala D.S., Das S., Maji S.K., Kumar A. (2017). Differential copper binding to alpha-synuclein and its disease-associated mutants affect the aggregation and amyloid formation. Biochim. Biophys. Acta Gen. Subj..

